# Organic matter source and degradation as revealed by molecular biomarkers in agricultural soils of Yuanyang terrace

**DOI:** 10.1038/srep11074

**Published:** 2015-06-05

**Authors:** Fangfang Li, Bo Pan, Di Zhang, Xiaolei Yang, Hao Li, Shaohua Liao, Abdul Ghaffar, Hongbo Peng, Baoshan Xing

**Affiliations:** 1 Faculty of Environmental Science & Engineering, Kunming University of Science & Technology, Kunming, 650500, China; 2 Stockbridge School of Agriculture, University of Massachusetts, Amherst, MA 01003.

## Abstract

Three soils with different tillage activities were collected and compared for their organic matter sources and degradation. Two soils (TD and TP) with human activities showed more diverse of chemicals in both free lipids and CuO oxidation products than the one (NS) without human activities. Branched alkanoic acids only accounted for less than 5% of lipids, indicating limited microbial inputs in all three investigated soils. The degradation of lignin in NS and TD was relatively higher than TP, probably because of the chemical degradation, most likely UV light-involved photodegradation. Lignin parameters obtained from CuO oxidation products confirmed that woody gymnosperm tissue (such as pine trees) may be the main source for NS, while angiosperm tissues from vascular plant may be the predominant source for the lignins in TD and TP. Analysis of BPCAs illustrated that BC in NS may be mainly originated from soot or other fossil carbon sources, whereas BC in TD and TP may be produced during corn stalk and straw burning. BC was involved in mineral interactions for TD and TP. The dynamics of organic matter needs to be extensively examined for their nonideal interactions with contaminants.

Soil organic matter (SOM) plays a key role in carbon cycling in the terrestrial ecosystem and its carbon storage is about twice higher than that in the atmosphere^1^,^2^. Hence, it is essential to understand the mechanism of SOM carbon sequestration in terrestrial systems. Up to date, the following processes were generally involved in SOM carbon sequestration: (1) physical protection (aggregates or microspores of organic matter); (2) chemical stabilization (interactions between SOM and soil mineral particles); (3) biochemical stabilization (recalcitrant SOM compounds)^3^,^4^. Different models have been developed to quantify carbon dynamics in terrestrial systems. For example, Parton *et al.* developed the Century model to estimate SOC storage changes^5^. Jenkinson and Coleman reported the Rothamsted Carbon model to simulate the turnover of SOC with different soil texture and plant types^6^. Most of the models used the bulk carbon content of soil particles as the major input parameter. The major hypothesis of most model concepts is that SOM could be treated as a homogeneous matrix, and the degradation rate stays constant as degradation proceeds. However, this hypothesis is not valid in most systems, because SOM is well-known to be a heterogeneous and complex mixture. For example, many studies have demonstrated that different compositions of SOM have different stabilities^7^. Although lignin is considered as a SOM precursor with long residence time compared to other SOM precursors, Amelung *et al.* reported that lignin would be more susceptible to degrade than polysaccharides in the presence of soil minerals^8^. Feng and Simpson also found that some aliphatic matters would be preferentially preserved compared to lignin compounds through selective sorption by clay minerals^9^.

Another important environmental function of SOM is that pollutant fate is largely dependent on their interactions with SOM. Recent studies suggested that different SOM fractions interact differently with pollutants, depending on their hydrophobicity, functional groups and maturity^10^,^11^. It is thus important to realize that the behavior of individual SOM component should be incorporated in SOM behavior studies for a better understanding of their roles in carbon cycling and pollutant fate. However, conventional chemical characterization techniques based on bulk properties, such as elemental compositions, NMR, FTIR, could not identify the sources and degradation status of different SOM compositions. Molecular-level characterization methods such as molecular biomarkers, provided a useful way to understand the molecular-level composition and sources of SOM^12^. Biomarkers are structurally unique molecules that retain carbon skeleton information of their parent precursor molecules during SOM evolution and environmental processes. It is therefore used as a powerful tracer for SOM^13^. The relationships between biomarkers and SOM sources/properties were systematically reviewed in previous studies^8^,^14^.

Previous studies have used biomarker method to examine SOM turnover and selective preservation in forest, grassland soils and sediments in response to climate change^15^ and vegetation alteration^7^. Vertical distributions and degradation stage of SOM in forest and grassland soils have also been reported^16^. Although SOM turnover is very fast in human-involved soils (typically tillaged soils), studies seldom focus on soils in these areas. SOM behavior in tillaged soils is poorly known^17^. In China, the area of arable soils is very large. According to the second national land survey, the areas of national arable soils were 121.7 million hectares, accounting for 12.8% in total national land [China statistical yearbook. http://www.stats.gov.cn/tjsj/ndsj/2013/indexch.htm, (2013) (Date of access: 07/03/2015)]. Therefore, SOM in tillaged soils play a significant role in global carbon cycling.

Yuanyang terrace area is located in Honghe, Yunnan, Southwestern China. This area is famous for the indigenous people with rich ecological knowledge, and the conservation of the unique terraced agricultural landscapes. This rice terrace landscape has been used for over 1000 years with a rich biodiversity in the nearby forest. The temperate and rainy climate promoted active SOM behavior in this area. In this study, soils samples were taken in areas with different agricultural activities. Solvent extraction was used to obtain solvent-extractable lipids and CuO oxidation was applied to quantify lignin-derived phenol organics. Molecular biomarkers for black carbon were also determined and compared. Understanding SOM behavior in this area will definitely provide important information for soil management and to evaluate carbon cycling in human-affected ecological systems.

## Results and Discussion

### Composition and source of solvent extractable free lipids

Both human activity-impacted soils (TD and TP) showed higher organic contents in comparison to NS, as suggested by their 5 times higher C contents than NS (Table 1). After carbon-normalization, free lipid content of NS was the highest and its CuO oxidation products were the lowest among the three soils samples.

**Figure 1:**
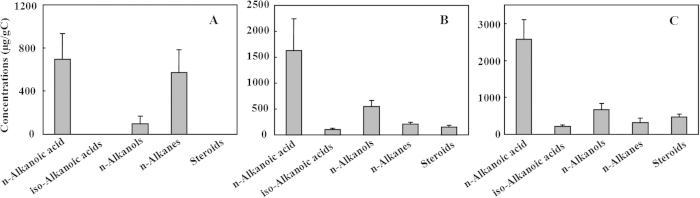
Major chemical compositions in solvent extractable free lipids. Un-disturbed natural soil (NS), soil from dry-farming land (TD), soil from terrace paddy field (TP) are separately presented in panels **A, B** and **C,** respectively.

The chemicals detected in the solvent extractable free lipids contained n-alkanols, n-alkanoic acids, iso- alkanoic acids, n-alkanes, and steroids as presented in Supplementary Figure S1. Monoacylglycerides and carbohydrates were also detected as minor components. The first observation from free lipids is that the soils with human activities showed more diverse of chemicals (Fig. 1). Human activities may have introduced various origins of biomass into the soil during the crop rotation, and application of composts^18^,^19^. The second observation is that aliphatic lipids were the dominating components in the free lipids of three soils, accounting for 51–58.7% of the detected chemicals in free lipids. n-Alkanoic acids are the most abundant fraction occupying 50.9–68.0% of aliphatic lipids. It has been previously reported that n-alkanoic acids tended to accumulate in more acidic soils while n-alkanes was enhanced in more alkaline soils^18^.

**Figure 2:**
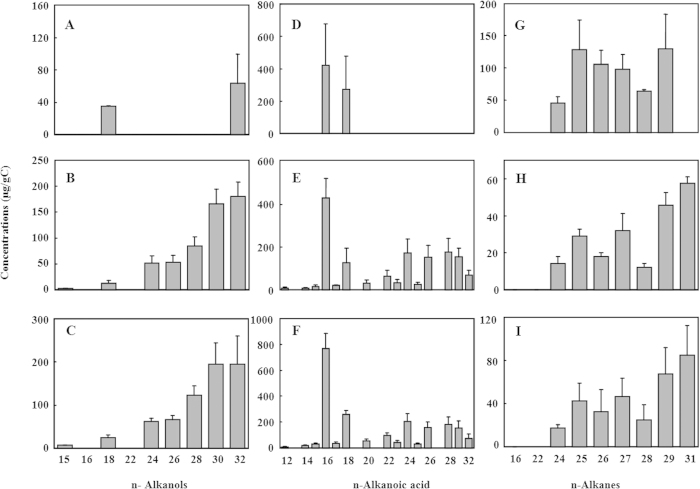
Distribution of n-alkanoic acids (**A**, **B**, and **C**), n-alkanols (**D**, **E**, and **F**), n-alkanes (**G**, **H**, and **I**) of solvent extraction in three soils. Panels **A**, **D** and **G** are for un-disturbed natural soil (NS), panels **B**, **E** and **H** for soil from dry-farming land (TD), and **C**, **F** and I for soil from terrace paddy field (TP).

The third observation is that n-alkanoic acids in the range of C_12_-C_32_ showed a significant even over odd dominance (Fig. 2D–F) and the highest two abundances were observed at C_16_ and C_18_. n-Alkanols in the range of C_15_-C_32_ also exhibited a preference of the even numbered molecules (Fig. 2A–C), and the highest abundance was C_30_ and C_32_ n-alkanol. Although the number of detected chemicals was less abundant in NS than in the other two soils, the trend was the same for the three samples. The even-over-odd predominance in the solvent extractable free lipids indicated a major input of lipids derived from higher plant waxes^20^. The solvent extracts also included two C_16_ and C_18_ monoacylglycerides, which may be attributed to the constituents of plant or microbial membranes^20^. Short-chain alkanes, alkanoic acid and diacids, iso-alkanoic acid mainly are the sources of microorganism^21^. However, only three branched alkanoic acids in the TD and TP soils were detected, namely iso-tetradecanoic acid, iso-hexadecanoic acid, and iso-heptadecanoic acid (Table S1). These branched alkanoic acids only accounted for less than 5% of aliphatic lipids, indicating that microbial inputs were present as minor components in all soils. Thus, plant inputs should be the main source of free lipids in all three soils. For TD and TP, biomarkers of steroids and phenols, the detected campesterol, stigmasterol and β-sitosterol, contributed to over 90% of total steroids and were the common steroids in the waxes of higher plants^20^. This observation also suggested the importance of plant input. Aliphatic/cyclic lipid ratio is a general parameter that indicates the degradation stage of SOM in soils^22^. The aliphatic/cyclic lipids ratios were 15.4 ± 0.3 and 7.2 ± 0.5 for TD and TP, respectively. The lower aliphatic/cyclic lipids ratio suggested that cyclic lipids were better preserved in TP, which is in a reduction environment.

Among the three tested soils, NS always showed low abundance of chemical compounds in comparison to other two soils. This phenomenon could not be attributed to the low organic matter content of NS and thus below the detection limits, because the concentrations for the detected chemicals were in the same range as those in TD and TP. Again, this is because of the less intense micro-biological activities in NS as suggested by the lack of microorganism-related biomarkers.

### Sources and degradation status of lignin as suggested by CuO oxidation products

**Figure 3:**
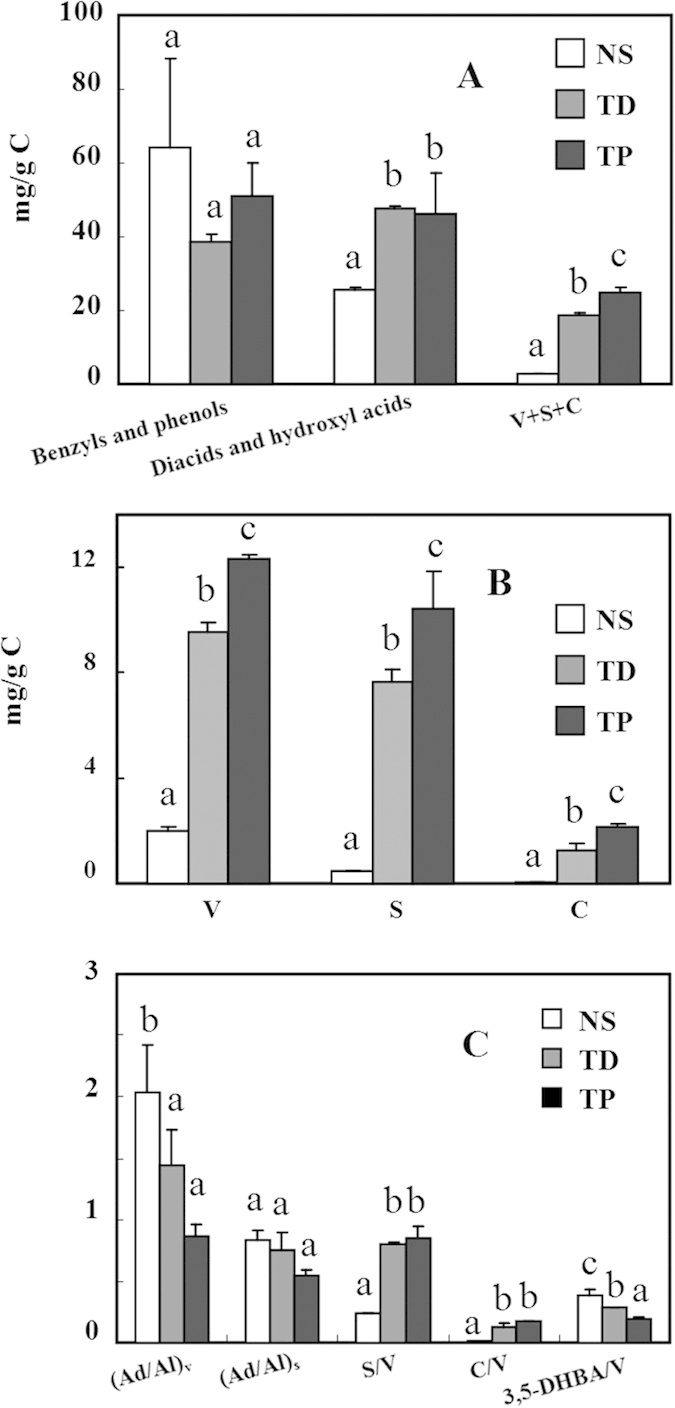
Biomarker concentrations and their comparison in CuO oxidation products of three soils. **A**: Carbon-normalized concentrations of main biomarkers in CuO oxidation products; **B**: Carbon-normalized concentrations of vanillyl (V), syringy (S) and cinnamyl (**C**) phenols; C: Degradation parameters of CuO products. (Ad/Al)v: ratio of vanillic acid to vanillin in the vanillyl unit; (Ad/Al)s: ratio of syringic acid to syringaldehyde in the syringyl unit; (Ad/Al)p: ratio of *p*-hydroxybenzoic acid to *p*-hydroxybenzaldehyde; 3,5-DHBA/V: ratio of 3,5-dihydroxybenzoic acid to vanillyl unit.

Major products of CuO oxidation in the three investigated soils were benzyls, phenols, short-chain n-alkanedioic acids and hydroxyl acids (Figure S2). Again, NS showed less abundance of chemicals in CuO oxidation products (Table S2). Five lignin parameters, namely S (syringyls), V (vanillyls), C (cinnamyls), S/V, and C/V, contain important information regarding plant-sources of SOM^23^. The amounts of V and S were much higher than C (Fig. 3B) in TD and TP, indicating their dominated contribution of woody plant tissue^23^,^24^. S/V value is widely used to differentiate the relative contributions of gymnosperm or angiosperm (S/V > 0.6)^25^. This value varied in the range of 0.23–0.84 in the three soils with the highest in TP. Angiosperm tissues from vascular plant may be the predominant source for the lignins in TD and TP as suggested by their S/V values above 0.6. Gymnosperm tissue may be the main source for NS as suggested by it low S/V value (0.23), which is consistent with the dominant pine species in the sampling area.

**Figure 4:**
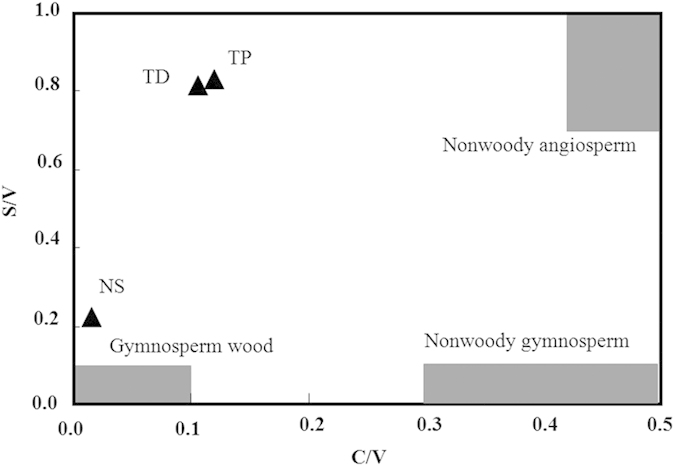
Lignin source parameters of the three investigated soils. C/V is calculated as the ratio of cinnamyl/vanillyl phenols. S/V is syringyl/vanillyl phenols. The plot of S/V vs. C/V is modified from reference^26^.

According to the plot of S/V vs. C/V (Fig. 4), lignin-derived SOM of these soils showed mixtures of different sources, including at least two different types of plant tissues (nonwoody angiosperm and woody gymnosperm). Lignin phenol vegetation index (LPVI) is another useful parameter to identify lignin sources^24^, and is calculated as s*(s + 1)/(v + 1) + 1)*((c + 1)/(v + 1) + 1) (in which s = 100*S/(S + V + C), c = 100*C/(S + V + C), and v = 100*V/(S + V + C)). The resulted LPVI values were 67.7 ± 12.0 for TD, and 96.8 ± 10.4 for TP. This result again suggested that woody angiosperm tissues (LPVI in the range of 67-415) are a significant contribution to the lignin-derived SOM in TD and TP. According to the field investigation, the local farmers either burnt the biomass in the farm land, or use the straws as forage of livestock. Thus, nonwoody-materials seldom accumulated in TD and TP. In addition, woody angiosperm-derived SOM in the original soil before cultivation may be protected in the soil particles. Some fresh organic matter carried in the irrigation water may also contribute to the woody angiosperm-derived SOM in TD and TP. The LPVI value of NS was 5.7 ± 0.4, which is consistent with the C/V value, suggesting the important input of woody gymnosperm (such as pine trees).

Lignin parameters of VSC, (Ad/Al)_v_, (Ad/Al)_s_, and 3,5-DHBA/V are generally used to describe the extent of plant organic matter degradation^23^. The yields of VSC (V + S + C) ranged from 2.5 ± 0.1 to 24.9 ± 1.5 mg/g C (Fig. 3C). This yield was the lowest for NS among three soils, and the highest for TP. (Al/Ad)_v_ ratios were in the range of 0.87 ± 0.09–2.04 ± 0.39 with the trend of TP < TD < NS (Fig. 3C). 3,5-Dihydroxybenzoic acid (3,5-DHBA) in the CuO oxidation products was derived from tannin and mainly accumulated in decaying cells. The higher value of 3,5-DHBA over vanillyls (3,5-DHBA/V) suggested increased degradation^27^,^28^. Therefore, combining the evidence of the degradation parameters ((Al/Ad)_v_, (Al/Ad)_s_, 3,5-DHBA/V)^29^, the degradation level of lignin in NS and TD was higher than that of TP. TP is often in the sub-aqueous environment, facilitating the preservation of the lignin composition under reducing conditions^30^. Meanwhile, TD was more subjected to photochemical degradation than the NS. Previous investigations have suggested that photodegradation of SOM could be very significant^31^, accounting for up to 90% of summer mid-day CO_2_ fluxes^32^. Although the organic components in soil are not readily exposed to UV light due to the protection by litter layer and soil minerals, human activities of crop residue removal, cultivation or harvest as well as soil erosion will greatly enhance the chance of photodegradation^31^. This is especially true for arid and semi-arid regions^33^,^34^,^35^. The sampling area receives intensive UV irradiation and could provide important energy source for photochemical degradation, which may be an important driving force for SOM degradation.

### Composition and source of black carbon as suggested by BPCAs method

Benzene-polycarboxylic acids (BPCAs) were used as biomarkers to describe BC in agricultural soils^36^,^37^. Major biomarkers of BC were detected in the three investigated soils as listed in Table 2. BC contents were also measured using the conventional chemical thermal oxidation (CTO) for comparison. These two methods provided different information regarding BC properties. CTO method may be more suited for obtaining the total amount of soot/graphitic BC^38^. NS had more abundant BC, around 7 times higher than TD and TP when normalized by TOC. BPCA method detects chemicals of a wide range of combustion continuum, which enables the analysis of BC properties based on the distribution of these chemicals^38^. The ratios of B5CA/B6CA and B6CA/B4CA could be used to trace BC sources. B5CA/B6CA values in the range of <0.8, 0.8–1.4, and 1.3–1.9 were attributed to wood fuel domestic fires, grass fires, and forest ground fires, respectively, while B6CA/B4CA values of <2, >2, and >7 were from grass fire, urban soils and fossil, respectively^39^,^40^. With the low value of B5CA/B6CA and high value of B6CA/B4CA, we speculated that BC in NS may be mainly originated from soot or other fossil carbon sources. The same parameters suggested that the BC in TD and TP may be produced during the grass fire (corn stalk and straw fire). The deposition of soot is also expected in TD and TP, but contributed less significantly because of the large amount of BC input from grass fire, or the soot were altered (mostly oxidized) due to the intense tillage activities. The yield of B6CA and B5CA was the highest among the 6 detected BPCAs in all soils. For example, B6CA accounted for 62.6%, 43.2%, and 41.7% of BPCAs in NS, TD and TP, respectively. The high content of B6CA and B5CA indicated high degree of BC condensation in these samples^41^. The higher degree of BC condensation in NS is consistent with its soot origin.

**Figure 5:**
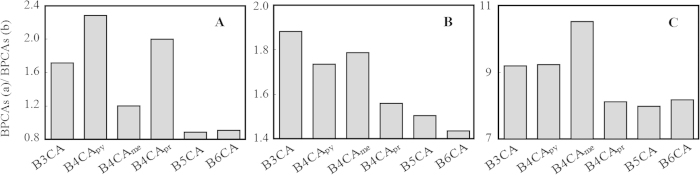
The ratios BPCA concentrations after (BPCAs (**a**)) and before (BPCAs (**b**)) mineral removal.

BPCAs were also compared for soil sample before and after mineral removal. The sum of BPCAs in TP and TD increased greatly after mineral removal, suggesting that BC was involved in mineral interactions for these two soils. Previous studies have suggested the BC may be physically included in the soil aggregates^42^,^43^,^44^ and the authors proposed the possibility of BC interacting with mineral particles. However, it should be noted that these studies are all based on density fractionation of soil particles. Thus, aggregate-occluded and organo-mineral complexation could not be distinguished^42^. According to the best of the authors’ knowledge, this is the first study incorporating mineral removal in BC biomarker (BPCAs) study. The extent of mineral complexation could also be compared when analyzing the ratios between individual BPCA concentrations after and before mineral removal (Fig. 5). Clearly, the extent of BPCA complexation was higher for B3CA and B4CA (represent small aromatic cluster size) than B5CA and B6CA (represent aromatic condensed BC). This result suggested that aromatic cluster with small size could form organo-mineral complexes more easily than the aromatic condensed ones. Previous studies suggested that BC may be associated with mineral particles through surface complexation-ligand exchange mechanism^45^. The less condensed BC may contain partial oxidation fractions, which facilitates their interactions with mineral surface. BPCA increase after HF treatment was not observed in NS, probably because the condense soot hardly incorporated in soil mineral compositions.

## Conclusions

Soil without human activity showed less abundance of biomarker chemicals in comparison to the soils with human activity. Human activities may have introduced or accelerated SOM turnover in soils. The higher degradation extent of SOM in NS and TD may be related to chemical degradation, and SOM was better preserved in the reduction environment of TP. Organic fractions showed some woody angiosperm tissues in tillage soils, which may be preserved in the organo-mineral complexes from the original soil before tillage. This mineral protection was also involved in BCs. Unlike common BC analysis methods, biomarkers (BPCAs) identified different BC fractions and origins. Aromatic cluster with small sizes in BCs could form organo-mineral complexes more easily than the aromatic condensed ones. BCs in NS was mostly from soot or other fossil carbon sources, and hardly complexed with mineral particles. Human activities significantly altered SOM compositions and properties, and thus extended work is demanded to relate the dynamics of carbon cycling to their nonideal interactions with contaminants.

## Methods

### Sample collection and preparation

Three soil samples, which are natural soils (NS, without tillage behavior), a terrace paddy soil (TP) and a terrace dry farming land soil (TD), were collected in Yuanyang (102°27′–103°13′E, 22°49′–23°19′N), Yunnan province. TP and TD have been tilled for around 50 years according to the field investigation with the local managers. All the samples were run in duplicate and the organic elemental contents are summarized in Table 1.

### Solvent extraction of free lipids

Free lipids were extracted according to the protocols described in previous studies^12^. In brief, 20 g soil samples were sonicated sequentially in 30 mL of dichloromethane, dichloromethane: methanol (1:1; v/v), and methanol for 15 min in each solvent. The extracts were centrifuged at 2500 rpm for 30 min. The supernatants were filtered through glass fiber filters (Whatman GF/A 1.6 um), concentrated by rotary evaporation, and then completely dried in 2 mL glass vials under N_2_ purge. The extract yields were determined by weighing the dry residue.

### CuO oxidation

The residues of the above-mentioned lipid extraction were air-dried and then subjected to CuO oxidation to release lignin-derived phenols^12^. Briefly, 2 g of soil residues were mixed with 1 g CuO (pre-extracted with dichloromethane), 100 mg ammonium iron sulfate hexahydrate [Fe(NH_4_)_2_(SO4)_2_.6H_2_O] and 15 mL of 2 M NaOH in teflon-lined bombs at 170 °C for 4.5 h. The extracts were acidified to pH 1 with 6 M HCl, and kept for 1 h at room temperature in the dark to minimize cinnamic acid reactions. After centrifuged at 2500 rpm for 30 min, the supernatants were liquid–liquid extracted with diethyl ether. The ether extracts were concentrated by rotary evaporation, transferred to 2 mL glass vials and dried under the purge of pure N_2_.

### Black carbon biomarker extraction

In order to investigate the interactions of BC with mineral particles, soil particles before and after HF treatment were extracted for BPCA analysis. In brief, 0.5 g of the original soil sample was digested using 10 mL of 4 M trifluoroacetic acid (TFA) at 105 °C for 4 h. After cooling, the residue was rinsed several times with deionized water by filtration through a glass fiber filter (Whatman GF/A 1.6 μm), and dried at 40 °C for 3 h. The residue was then transferred to Teflon-lined bombs. Two mL of 65% HNO_3_ were added in the bombs and reacted at 170 °C for 8 h. After cooling, the mixture was filtered by an ash-less cellulose filter. The digestion solution was diluted 5 times and then treated using cation exchange resin (Dowex 50 WX8, 200-400 mesh). The treated aqueous samples were freeze-dried for further processing.

### Derivatization and GC-MS analysis

The extracts of free lipid extraction, CuO oxidation, and BC biomarkers were separately dissolved and then trimethylsilyl (TMS) derivatized. All the derivatized solutions were subjected to GC–MS analyses (Agilent, 7890 A GC equipped with 5975C quadrupole mass selective detector). TMS derivatives of n-heptadecanoic acid and ergosterol were used as external quantification standards for free lipid fractions and the TMS derivatives of vanillic acid were used as the standard for CuO oxidation products.

More detailed experimental conditions are presented in supplementary information.

## Additional Information

**How to cite this article**: Li, F. *et al.* Organic matter source and degradation as revealed by molecular biomarkers in agricultural soils of Yuanyang terrace. *Sci. Rep.* **5**, 11074; doi: 10.1038/srep11074 (2015).

## Supplementary Material

Supplementary Information

## Figures and Tables

**Table 1:** Organic elemental contents and extraction yields of the investigated soils.

	**N (%)**	**C (%)**	**H (%)**	**S (%)**	**C/H**^a^	**C/N**^a^	**Free lipids (mg/g C)**	**CuO oxidation products (mg/g C)**
NS^b^	0.06	0.45	0.97	0.08	0.04	8.49	17.0 ± 0.3	78.7 ± 8.6
TD^b^	0.19	1.94	1.02	0.04	0.16	12.0	7.06 ± 0.6	154 ± 8.4
TP^b^	0.18	1.93	1.01	0.04	0.16	12.5	11.4 ± 0.3	178 ± 8.4

^a^atom-number based values.

^b^NS: Un-disturbed natural soil; TD: Soil from dry-farming land; TP: Soil from terrace paddy field.

**Table 2:** Black carbon biomarkers, BPCAs in the investigated soils.

**(mg/g C)**	**B3CA**	**B4CA_py_**	**B4CA_me_**	**B4CA_pr_**	**B5CA**	**B6CA**	**Sum**	**5/6**	**6/4**	**CTO**
NS	0.7 ± 0.3	0.7 ± 0.1	1.0 ± 0.1	1.6 ± 0.0	11.1 ± 1.9	25.4 ± 3.4	40.5	0.44	7.7	280 ± 10
NS-HF	1.2 ± 0.1	1.6 ± 0.1	1.2 ± 0.3	3.2 ± 0.1	9.8 ± 0.6	23.1 ± 4.9	40.1	0.42	3.9	270 ± 7
TD	1.7 ± 0.1	3.4 ± 0.2	2.8 ± 0.2	6.8 ± 0.6	25.0 ± 2.2	30.2 ± 2.3	69.9	0.83	2.3	43 ± 2
TD-HF	3.2 ± 0.1	5.9 ± 0.1	5.0 ± 0.1	10.6 ± 0.4	37.6 ± 2.2	43.3 ± 2.6	105.6	0.87	2.0	39 ± 3
TP	1.6 ± 0.2	3.0 ± 0.5	2.5 ± 3.6	5.8 ± 2.1	23.6 ± 6.4	26.2 ± 5.0	62.7	0.90	2.3	39 ± 2
TP-HF	14.7 ± 2.0	27.7 ± 0.7	26.3 ± 1.2	47.1 ± 1.2	188.2 ± 2.3	214.0 ± 4.9	518	0.88	2.1	26 ± 2

The suffix of “-HF”: Soil samples after mineral fraction removal

B3CA: Trimellitic acid

B4CA_py_: Pyromellitic Acid

B4CA_me_: Mellophanic acid

B4CA_pr_: Prehnitic acid

B5CA: Benzenepenta-carboxylic acid

B6CA: Mellitic acid

5/6: B5CA / B6CA

6/4: B6CA / B4CA

CTO: Chemical thermal oxidation.
