# Supplementary material for: Organic matter source and degradation as revealed by molecular biomarkers in agricultural soils of Yuanyang terrace
Source: Sci Rep. 2015 Jun 5;5:11074. doi: 10.1038/srep11074 (PMC4457134; doi:10.1038/srep11074)
Supplement: Supplementary Information [file srep11074-s1.doc]

Supplementary Information

**Organic matter source and degradation as revealed by molecular biomarkers in agricultural soils of Yuanyang terrace**

Fangfang Li ^1^, Bo Pan ^1^*, Di Zhang^1^, Xiaolei Yang^1^, Hao Li^1^, Shaohua Liao^1^, Abdul Ghaffar ^1^, Hongbo Peng^1^, Baoshan Xing^2^

1. Faculty of Environmental Science & Engineering, Kunming University of Science & Technology, Kunming, China, 650500

2. Stockbridge School of Agriculture, University of Massachusetts, Amherst, MA 01003

*Corresponding author: Dr. Pan, phone/fax: 86-871-65170906; e-mail: [panbocai@gmail.com](mailto:panbocai@gmail.com)

**Table S1. Major compounds identified in solvent-extractable free lipid in three** soils

| **Compound name** | **MW (g/mol)** | **Composition** | **Concentrations (μg/g C)** | |
| --- | --- | --- | --- | --- |
|  |  |  | **NS** |  |
| n-Alkanoic acids |  |  |  |  |
| n-Nonanoic acid | 158.0 | C9H18O2 | 0.0 |  |
| n-Decanoic acid | 172.0 | C10H20O2 | 0.0 |  |
| n-Dodecanoic acid | 200.0 | C12H24O2 | 0.0 |  |
| n-Tridecanoic acid | 214.0 | C13H26O2 | 0.0 |  |
| n-Tetradecanoic acid | 228.0 | C14H28O2 | 0.0 |  |
| n-Pentadecanoic acid | 242.0 | C15H30O2 | 0.0 |  |
| n-Hexadecenoic acid(C16:1) | 254.0 | C16H30O2 | 0.0 |  |
| n-Hexadecenoic acid(C16:1) | 254.0 | C16H30O2 | 0.0 |  |
| n-Hexadecanoic acid | 256.0 | C16H32O2 | 420.4 |  |
| n-Heptadecanoic acid | 270.0 | C17H34O2 | 0.0 |  |
| n-Octadecadienoic acid (C18:2) | 280.0 | C18H32O2 | 0.0 |  |
| n-Octadecenoic acid(C18:1) | 282.0 | C18H34O2 | 0.0 |  |
| n-Octadecenoic acid(C18:1) | 282.0 | C18H34O2 | 0.0 |  |
| n-Octadecanoic acid | 284.0 | C18H36O2 | 273.5 |  |
| n-Nonadecenoic acid(C19:1) | 296.0 | C19H36O2 | 0.0 |  |
| n-Nonadecanoic acid | 298.0 | C19H38O2 | 0.0 |  |
| n-Eicosanoic acid | 312.0 | C20H40O2 | 0.0 |  |
| n-Heneicosanoic acid | 326.0 | C21H42O2 | 0.0 |  |
| n-Docosenoic acid(C22:1) | 338.0 | C22H42O2 | 0.0 |  |
| n-Docosanoic acid | 340.0 | C22H44O2 | 0.0 |  |
| n-Tricosanoic acid | 354.0 | C23H46O2 | 0.0 |  |
| n-Tetracosanoic acid | 368.0 | C24H48O2 | 0.0 |  |
| n-Pentacosanoic acid | 382.0 | C25H50O2 | 0.0 |  |
| n-Hexacosanoic acid | 396.0 | C26H52O2 | 0.0 |  |
| n-Heptacosanoic acid | 410.0 | C27H54O2 | 0.0 |  |
| n-Octacosanoic acid | 424.0 | C28H56O2 | 0.0 |  |
| n-Triacontanoic acid | 452.0 | C30H60O2 | 0.0 |  |
| n-Dotriacontanoic acid | 480.0 | C32H64O2 | 0.0 |  |
| Total |  |  | 693.9 |  |
| Branched Alkanoic acids |  |  |  |  |
| iso-Pentadecanoic acid | 242.0 | C15H30O2 | 0.0 |  |
| iso-Hexadecanoic acid | 256.0 | C16H32O2 | 0.0 |  |
| iso-Heptadecanoic acid | 270.0 | C17H34O2 | 0.0 |  |
| iso-Heptadecanoic acid | 270.0 | C17H34O2 | 0.0 |  |
| Total |  |  | 0.0 |  |
| n-Alkanols |  |  |  |  |
| n-Pentadecanol | 228.0 | C15H32O | 0.0 |  |
| n-Hexadecanol | 242.0 | C16H34O | 0.0 |  |
| n-Octadecanol | 270.0 | C18H38O | 34.8 |  |
| n-Docosanol | 326.0 | C22H46O | 0.0 |  |
| n-Tetracosanol | 354.0 | C24H50O | 0.0 |  |
| n-Hexacosanol | 382.0 | C26H54O | 0.0 |  |
| n-Octacosanol | 410.0 | C28H56O | 0.0 |  |
| n-Triacontanol | 438.0 | C30H62O | 0.0 |  |
| n-Dotriacontanol | 466.0 | C32H66O | 63.6 |  |
| Total |  |  | 98.4 |  |
| n-Alkanes |  |  |  |  |
| n-Hexadecane | 226.0 | C16H34 | 0.0 |  |
| n-Docosane | 310.0 | C22H46 | 0.0 |  |
| n-Tetracosane | 338.0 | C24H50 | 45.3 |  |
| n-Pentacosane | 352.0 | C25H52 | 127.7 |  |
| n-Hexacosane | 366.0 | C26H54 | 105.9 |  |
| n-Heptacosane | 380.0 | C27H56 | 97.8 |  |
| n-Octacosane | 394.0 | C28H58 | 64.1 |  |
| n-Nonacosane | 408.0 | C29H60 | 129.0 |  |
| n-Hentriacontane | 436.0 | C31H64 | 0.0 |  |
| Total |  |  | 569.9 |  |
| Aliphatic lipids total |  |  | 1362.2 |  |
| Steroids |  |  | 0.0 |  |
| Cholesterol | 386.0 | C27H46O | 0.0 |  |
| Campesterol | 400.0 | C28H48O | 0.0 |  |
| Stigmasterol | 412.0 | C29H48O | 0.0 |  |
| .beta.-Sitosterol | 414.0 | C29H50O | 0.0 |  |
| Total |  |  | 0.0 |  |
| Carbohydrates |  |  |  |  |
| L(+)Arabinose |  |  | 0.0 |  |
| L(+)Rhamnose | 164.0 | C6H12O5 | 0.0 |  |
| D(-)Ribose |  |  | 0.0 |  |
| L(-)Fucose |  |  | 0.0 |  |
| Mannosan |  |  | 0.0 |  |
| Levoglucosan |  |  | 0.0 |  |
| D(+)Fucose |  |  | 0.0 |  |
| .alpha.-D(+)Xylose | 150.0 | C5H10O5 | 0.0 |  |
| .alpha.-D(+)Glucose | 180.0 | C6H12O6 | 0.0 |  |
| .beta.-D(+)Glucose | 180.0 | C6H12O6 | 0.0 |  |
| Trehalose | 342.0 | C12H22O11 | 0.0 |  |
|  |  |  | 0.0 |  |
| C16 Monoacylglyceride | 330 | C19H38O4 | 491.0 |  |
| C18 Monoacylglyceride | 358 | C21H42O4 | 514.9 |  |

| **Compound name** | **MW (g/mol)** | **Composition** | **Concentrations (μg/g C)** | |
| --- | --- | --- | --- | --- |
|  |  |  | **TD** |  |
| n-Alkanoic acids |  |  |  |  |
| n-Nonanoic acid | 158.0 | C9H18O2 | 11.3 |  |
| n-Decanoic acid | 172.0 | C10H20O2 | 0.0 |  |
| n-Dodecanoic acid | 200.0 | C12H24O2 | 7.9 |  |
| n-Tridecanoic acid | 214.0 | C13H26O2 | 0.0 |  |
| n-Tetradecanoic acid | 228.0 | C14H28O2 | 7.6 |  |
| n-Pentadecanoic acid | 242.0 | C15H30O2 | 16.4 |  |
| n-Hexadecenoic acid(C16:1) | 254.0 | C16H30O2 | 18.4 |  |
| n-Hexadecenoic acid(C16:1) | 254.0 | C16H30O2 | 15.3 |  |
| n-Hexadecanoic acid | 256.0 | C16H32O2 | 431.5 |  |
| n-Heptadecanoic acid | 270.0 | C17H34O2 | 19.4 |  |
| n-Octadecadienoic acid (C18:2) | 280.0 | C18H32O2 | 0.0 |  |
| n-Octadecenoic acid(C18:1) | 282.0 | C18H34O2 | 77.2 |  |
| n-Octadecenoic acid(C18:1) | 282.0 | C18H34O2 | 19.0 |  |
| n-Octadecanoic acid | 284.0 | C18H36O2 | 126.3 |  |
| n-Nonadecenoic acid(C19:1) | 296.0 | C19H36O2 | 0.0 |  |
| n-Nonadecanoic acid | 298.0 | C19H38O2 | 0.0 |  |
| n-Eicosanoic acid | 312.0 | C20H40O2 | 30.8 |  |
| n-Heneicosanoic acid | 326.0 | C21H42O2 | 0.0 |  |
| n-Docosenoic acid(C22:1) | 338.0 | C22H42O2 | 0.0 |  |
| n-Docosanoic acid | 340.0 | C22H44O2 | 62.6 |  |
| n-Tricosanoic acid | 354.0 | C23H46O2 | 34.1 |  |
| n-Tetracosanoic acid | 368.0 | C24H48O2 | 172.2 |  |
| n-Pentacosanoic acid | 382.0 | C25H50O2 | 25.7 |  |
| n-Hexacosanoic acid | 396.0 | C26H52O2 | 152.6 |  |
| n-Heptacosanoic acid | 410.0 | C27H54O2 | 0.0 |  |
| n-Octacosanoic acid | 424.0 | C28H56O2 | 177.9 |  |
| n-Triacontanoic acid | 452.0 | C30H60O2 | 154.1 |  |
| n-Dotriacontanoic acid | 480.0 | C32H64O2 | 68.5 |  |
| Total |  |  | 1628.9 |  |
| Branched Alkanoic acids |  |  |  |  |
| iso-Pentadecanoic acid | 242.0 | C15H30O2 | 52.1 |  |
| iso-Hexadecanoic acid | 256.0 | C16H32O2 | 24.7 |  |
| iso-Heptadecanoic acid | 270.0 | C17H34O2 | 14.8 |  |
| iso-Heptadecanoic acid | 270.0 | C17H34O2 | 10.6 |  |
| Total |  |  | 102.2 |  |
| n-Alkanols |  |  |  |  |
| n-Pentadecanol | 228.0 | C15H32O | 2.7 |  |
| n-Hexadecanol | 242.0 | C16H34O | 0.0 |  |
| n-Octadecanol | 270.0 | C18H38O | 12.8 |  |
| n-Docosanol | 326.0 | C22H46O | 0.0 |  |
| n-Tetracosanol | 354.0 | C24H50O | 51.3 |  |
| n-Hexacosanol | 382.0 | C26H54O | 53.2 |  |
| n-Octacosanol | 410.0 | C28H56O | 84.3 |  |
| n-Triacontanol | 438.0 | C30H62O | 166.5 |  |
| n-Dotriacontanol | 466.0 | C32H66O | 180.3 |  |
| Total |  |  | 551.2 |  |
| n-Alkanes |  |  |  |  |
| n-Hexadecane | 226.0 | C16H34 | 0.0 |  |
| n-Docosane | 310.0 | C22H46 | 0.0 |  |
| n-Tetracosane | 338.0 | C24H50 | 14.3 |  |
| n-Pentacosane | 352.0 | C25H52 | 29.1 |  |
| n-Hexacosane | 366.0 | C26H54 | 18.0 |  |
| n-Heptacosane | 380.0 | C27H56 | 31.8 |  |
| n-Octacosane | 394.0 | C28H58 | 12.3 |  |
| n-Nonacosane | 408.0 | C29H60 | 45.7 |  |
| n-Hentriacontane | 436.0 | C31H64 | 57.7 |  |
| Total |  |  | 208.8 |  |
| Aliphatic lipids total |  |  | 2491.2 |  |
| Steroids |  |  | 0.0 |  |
| Cholesterol | 386.0 | C27H46O | 12.5 |  |
| Campesterol | 400.0 | C28H48O | 21.7 |  |
| Stigmasterol | 412.0 | C29H48O | 66.9 |  |
| .beta.-Sitosterol | 414.0 | C29H50O | 60.8 |  |
| Total |  |  | 161.9 |  |
| Carbohydrates |  |  |  |  |
| L(+)Arabinose |  |  | 0.0 |  |
| L(+)Rhamnose | 164.0 | C6H12O5 | 0.0 |  |
| D(-)Ribose |  |  | 0.0 |  |
| L(-)Fucose |  |  | 0.0 |  |
| Mannosan |  |  | 0.0 |  |
| Levoglucosan |  |  | 0.0 |  |
| D(+)Fucose |  |  | 7.1 |  |
| .alpha.-D(+)Xylose | 150.0 | C5H10O5 | 0.0 |  |
| .alpha.-D(+)Glucose | 180.0 | C6H12O6 | 13.9 |  |
| .beta.-D(+)Glucose | 180.0 | C6H12O6 | 19.0 |  |
| Trehalose | 342.0 | C12H22O11 | 942.4 |  |
|  |  |  | 982.5 |  |
| C16 Monoacylglyceride | 330 | C19H38O4 | 366.9 |  |
| C18 Monoacylglyceride | 358 | C21H42O4 | 238.2 |  |

| **Compound name** | **MW (g/mol)** | **Composition** | **Concentrations (μg/g C)** | |
| --- | --- | --- | --- | --- |
|  |  |  | **TP** |  |
| n-Alkanoic acids |  |  |  |  |
| n-Nonanoic acid | 158.0 | C9H18O2 | 11.7 |  |
| n-Decanoic acid | 172.0 | C10H20O2 | 0.0 |  |
| n-Dodecanoic acid | 200.0 | C12H24O2 | 9.5 |  |
| n-Tridecanoic acid | 214.0 | C13H26O2 | 0.0 |  |
| n-Tetradecanoic acid | 228.0 | C14H28O2 | 19.2 |  |
| n-Pentadecanoic acid | 242.0 | C15H30O2 | 32.0 |  |
| n-Hexadecenoic acid(C16:1) | 254.0 | C16H30O2 | 62.4 |  |
| n-Hexadecenoic acid(C16:1) | 254.0 | C16H30O2 | 46.3 |  |
| n-Hexadecanoic acid | 256.0 | C16H32O2 | 768.1 |  |
| n-Heptadecanoic acid | 270.0 | C17H34O2 | 36.7 |  |
| n-Octadecadienoic acid (C18:2) | 280.0 | C18H32O2 | 53.6 |  |
| n-Octadecenoic acid(C18:1) | 282.0 | C18H34O2 | 228.1 |  |
| n-Octadecenoic acid(C18:1) | 282.0 | C18H34O2 | 42.0 |  |
| n-Octadecanoic acid | 284.0 | C18H36O2 | 257.6 |  |
| n-Nonadecenoic acid(C19:1) | 296.0 | C19H36O2 | 0.0 |  |
| n-Nonadecanoic acid | 298.0 | C19H38O2 | 0.0 |  |
| n-Eicosanoic acid | 312.0 | C20H40O2 | 55.0 |  |
| n-Heneicosanoic acid | 326.0 | C21H42O2 | 0.0 |  |
| n-Docosenoic acid(C22:1) | 338.0 | C22H42O2 | 0.0 |  |
| n-Docosanoic acid | 340.0 | C22H44O2 | 95.5 |  |
| n-Tricosanoic acid | 354.0 | C23H46O2 | 44.2 |  |
| n-Tetracosanoic acid | 368.0 | C24H48O2 | 204.6 |  |
| n-Pentacosanoic acid | 382.0 | C25H50O2 | 30.1 |  |
| n-Hexacosanoic acid | 396.0 | C26H52O2 | 156.9 |  |
| n-Heptacosanoic acid | 410.0 | C27H54O2 | 0.0 |  |
| n-Octacosanoic acid | 424.0 | C28H56O2 | 183.2 |  |
| n-Triacontanoic acid | 452.0 | C30H60O2 | 155.2 |  |
| n-Dotriacontanoic acid | 480.0 | C32H64O2 | 73.0 |  |
| Total |  |  | 2565.1 |  |
| Branched Alkanoic acids |  |  |  |  |
| iso-Pentadecanoic acid | 242.0 | C15H30O2 | 90.0 |  |
| iso-Hexadecanoic acid | 256.0 | C16H32O2 | 70.2 |  |
| iso-Heptadecanoic acid | 270.0 | C17H34O2 | 28.8 |  |
| iso-Heptadecanoic acid | 270.0 | C17H34O2 | 26.9 |  |
| Total |  |  | 216.0 |  |
| n-Alkanols |  |  |  |  |
| n-Pentadecanol | 228.0 | C15H32O | 7.1 |  |
| n-Hexadecanol | 242.0 | C16H34O | 0.0 |  |
| n-Octadecanol | 270.0 | C18H38O | 24.9 |  |
| n-Docosanol | 326.0 | C22H46O | 0.0 |  |
| n-Tetracosanol | 354.0 | C24H50O | 61.8 |  |
| n-Hexacosanol | 382.0 | C26H54O | 66.8 |  |
| n-Octacosanol | 410.0 | C28H56O | 123.2 |  |
| n-Triacontanol | 438.0 | C30H62O | 195.1 |  |
| n-Dotriacontanol | 466.0 | C32H66O | 194.6 |  |
| Total |  |  | 673.4 |  |
| n-Alkanes |  |  |  |  |
| n-Hexadecane | 226.0 | C16H34 | 0.0 |  |
| n-Docosane | 310.0 | C22H46 | 0.0 |  |
| n-Tetracosane | 338.0 | C24H50 | 17.4 |  |
| n-Pentacosane | 352.0 | C25H52 | 42.5 |  |
| n-Hexacosane | 366.0 | C26H54 | 32.4 |  |
| n-Heptacosane | 380.0 | C27H56 | 46.3 |  |
| n-Octacosane | 394.0 | C28H58 | 25.1 |  |
| n-Nonacosane | 408.0 | C29H60 | 67.4 |  |
| n-Hentriacontane | 436.0 | C31H64 | 84.8 |  |
| Total |  |  | 315.8 |  |
| Aliphatic lipids total |  |  | 3770.2 |  |
| Steroids |  |  | 0.0 |  |
| Cholesterol | 386.0 | C27H46O | 44.1 |  |
| Campesterol | 400.0 | C28H48O | 60.3 |  |
| Stigmasterol | 412.0 | C29H48O | 194.0 |  |
| .beta.-Sitosterol | 414.0 | C29H50O | 224.5 |  |
| Total |  |  | 522.8 |  |
| Carbohydrates |  |  |  |  |
| L(+)Arabinose |  |  | 0.0 |  |
| L(+)Rhamnose | 164.0 | C6H12O5 | 0.0 |  |
| D(-)Ribose |  |  | 0.0 |  |
| L(-)Fucose |  |  | 0.0 |  |
| Mannosan |  |  | 0.0 |  |
| Levoglucosan |  |  | 0.0 |  |
| D(+)Fucose |  |  | 14.8 |  |
| .alpha.-D(+)Xylose | 150.0 | C5H10O5 | 0.0 |  |
| .alpha.-D(+)Glucose | 180.0 | C6H12O6 | 28.9 |  |
| .beta.-D(+)Glucose | 180.0 | C6H12O6 | 16.7 |  |
| Trehalose | 342.0 | C12H22O11 | 1695.2 |  |
|  |  |  | 1755.7 |  |
| C16 Monoacylglyceride | 330 | C19H38O4 | 742.6 |  |
| C18 Monoacylglyceride | 358 | C21H42O4 | 605.1 |  |

**Table S2. Major compounds identified in CuO oxidation productions from the three** investigated soils

| **Compound name** | **MW (g/mol)** | **Composition** | **Concentrations (μg/g C)** | |
| --- | --- | --- | --- | --- |
| **Benzyls and phenols** |  |  | **NS** |  |
| Benzoic acid | 122.0 | C7H6O2 | 793.8 |  |
| Benzeneacetic acid | 136.0 | C8H8O2 | 422.2 |  |
| hydroxyphenylacetic acid | 152.0 | C8H8O3 | 558.2 |  |
| Butylated Hydroxytoluene | 220.0 | C15H24O | 54167.8 |  |
| 4-Hydroxybenzeneacetic acid | 152.0 | C8H8O3 | 0.0 |  |
| p-Hydroxybenzaldehyde | 122.0 | C7H6O2 | 289.4 |  |
| p-Hydroxyacetophenone | 136.0 | C8H8O2 | 0.0 |  |
| m-Hydroxybenzoic acid | 138.0 | C7H6O3 | 1327.4 |  |
| p-Hydroxybenzoic acid | 138.0 | C7H6O3 | 576.4 |  |
| 3,5-Dihydroxybenzoic acid | 154.0 | C7H6O4 | 770.8 |  |
| Vanillin | 152.0 | C8H8O3 | 563.9 |  |
| Acetovanillone | 154.0 | C8H10O3 | 285.7 |  |
| Vanillic acid | 168.0 | C8H8O4 | 1149.3 |  |
| Syringaldehyde | 182.0 | C9H10O4 | 229.5 |  |
| Acetosyringone | 196.0 | C10H12O4 | 43.6 |  |
| Syringic acid | 198.0 | C9H10O5 | 192.8 |  |
| p-Coumaric acid | 164.0 | C9H8O3 | 32.7 |  |
| Ferulic acid | 194.0 | C10H10O4 | 0.0 |  |
| 1,2-Benzenedicarboxylic acid | 166.0 | C8H6O4 | 2440.9 |  |
| Total |  |  | 63844.3 |  |
|  |  | V | 1998.8 |  |
|  |  | S | 465.8 |  |
|  |  | C | 32.7 |  |
| Diacids and hydroxy acids |  |  |  |  |
| 2-Hydroxypropanoic acid | 90.0 | C3H6O3 | 467.1 |  |
| 2-Ketoisocaproic acid | 140.0 | C6H10O3 | 172.4 |  |
| 2-Methyl-2-hydroxypropanoic acid | 104.0 | C4H8O3 | 842.1 |  |
| 2-Methyl-2-hydroxybutanoic acid | 118.0 | C5H10O3 | 161.8 |  |
| 4-Methyl-2-hydroxypentanoic acid | 132.0 | C6H12O3 | 0.0 |  |
| 2-Hydroxy-2-pentenedioic acid | 146.0 | C5H6O5 | 0.0 |  |
| 3-Hydroxybutanoic acid | 104.0 | C4H8O3 | 177.2 |  |
| 2-Hydroxybutanedioic acid | 134.0 | C4H6O5 | 132.2 |  |
| 2-Hydroxypentanedioic acid | 148.0 | C5H8O5 | 151.7 |  |
| 2-Hydroxyhexanedioic acid | 162.0 | C6H10O5 | 95.4 |  |
| Ethanedioic acid | 90.0 | C2H2O4 | 3175.5 |  |
| Propanedioic acid | 104.0 | C3H4O4 | 614.6 |  |
| Succinic acid | 118.0 | C4H6O4 | 3299.5 |  |
| 2-Methylbutanedioic acid | 132.0 | C5H8O4 | 896.0 |  |
| Fumaric acid | 116.0 | C4H4O4 | 14327.0 |  |
| Methylmaleic acid | 130.0 | C5H6O4 | 578.8 |  |
| Heptanedioic acid | 160.0 | C7H12O4 | 0.0 |  |
| Octanedioic acid | 174.0 | C8H14O4 | 229.9 |  |
| Azelaic acid | 188.0 | C9H16O4 | 372.0 |  |
| Sebacic acid | 202.0 | C10H18O4 | 0.0 |  |
| Total |  |  | 25693.1 |  |

| **Compound name** | **MW (g/mol)** | **Composition** | **Concentrations (μg/g C)** | |
| --- | --- | --- | --- | --- |
| **Benzyls and phenols** |  |  | **TD** |  |
| Benzoic acid | 122.0 | C7H6O2 | 1042.4 |  |
| Benzeneacetic acid | 136.0 | C8H8O2 | 559.2 |  |
| hydroxyphenylacetic acid | 152.0 | C8H8O3 | 468.3 |  |
| Butylated Hydroxytoluene | 220.0 | C15H24O | 5861.9 |  |
| 4-Hydroxybenzeneacetic acid | 152.0 | C8H8O3 | 411.7 |  |
| p-Hydroxybenzaldehyde | 122.0 | C7H6O2 | 2335.0 |  |
| p-Hydroxyacetophenone | 136.0 | C8H8O2 | 844.1 |  |
| m-Hydroxybenzoic acid | 138.0 | C7H6O3 | 2274.5 |  |
| p-Hydroxybenzoic acid | 138.0 | C7H6O3 | 2727.1 |  |
| 3,5-Dihydroxybenzoic acid | 154.0 | C7H6O4 | 2725.4 |  |
| Vanillin | 152.0 | C8H8O3 | 3165.2 |  |
| Acetovanillone | 154.0 | C8H10O3 | 1814.2 |  |
| Vanillic acid | 168.0 | C8H8O4 | 4585.6 |  |
| Syringaldehyde | 182.0 | C9H10O4 | 3273.5 |  |
| Acetosyringone | 196.0 | C10H12O4 | 1909.0 |  |
| Syringic acid | 198.0 | C9H10O5 | 2464.7 |  |
| p-Coumaric acid | 164.0 | C9H8O3 | 1014.8 |  |
| Ferulic acid | 194.0 | C10H10O4 | 220.5 |  |
| 1,2-Benzenedicarboxylic acid | 166.0 | C8H6O4 | 984.9 |  |
| Total |  |  | 38681.9 |  |
|  |  | V | 9565.0 |  |
|  |  | S | 7647.2 |  |
|  |  | C | 1235.2 |  |
| Diacids and hydroxy acids |  |  |  |  |
| 2-Hydroxypropanoic acid | 90.0 | C3H6O3 | 1614.0 |  |
| 2-Ketoisocaproic acid | 140.0 | C6H10O3 | 1928.1 |  |
| 2-Methyl-2-hydroxypropanoic acid | 104.0 | C4H8O3 | 1792.1 |  |
| 2-Methyl-2-hydroxybutanoic acid | 118.0 | C5H10O3 | 223.9 |  |
| 4-Methyl-2-hydroxypentanoic acid | 132.0 | C6H12O3 | 214.9 |  |
| 2-Hydroxy-2-pentenedioic acid | 146.0 | C5H6O5 | 254.3 |  |
| 3-Hydroxybutanoic acid | 104.0 | C4H8O3 | 469.8 |  |
| 2-Hydroxybutanedioic acid | 134.0 | C4H6O5 | 840.4 |  |
| 2-Hydroxypentanedioic acid | 148.0 | C5H8O5 | 801.6 |  |
| 2-Hydroxyhexanedioic acid | 162.0 | C6H10O5 | 1179.9 |  |
| Ethanedioic acid | 90.0 | C2H2O4 | 2486.3 |  |
| Propanedioic acid | 104.0 | C3H4O4 | 77.5 |  |
| Succinic acid | 118.0 | C4H6O4 | 4165.4 |  |
| 2-Methylbutanedioic acid | 132.0 | C5H8O4 | 1919.8 |  |
| Fumaric acid | 116.0 | C4H4O4 | 23815.9 |  |
| Methylmaleic acid | 130.0 | C5H6O4 | 919.2 |  |
| Heptanedioic acid | 160.0 | C7H12O4 | 661.0 |  |
| Octanedioic acid | 174.0 | C8H14O4 | 1262.9 |  |
| Azelaic acid | 188.0 | C9H16O4 | 2513.4 |  |
| Sebacic acid | 202.0 | C10H18O4 | 460.9 |  |
| Total |  |  | 47601.4 |  |

| **Compound name** | **MW(g/mol)** | **Composition** | **Concentrations(μg/g C)** | |
| --- | --- | --- | --- | --- |
| **Benzyls and phenols** |  |  | **TP** |  |
| Benzoic acid | 122.0 | C7H6O2 | 1125.1 |  |
| Benzeneacetic acid | 136.0 | C8H8O2 | 543.6 |  |
| hydroxyphenylacetic acid | 152.0 | C8H8O3 | 346.3 |  |
| Butylated Hydroxytoluene | 220.0 | C15H24O | 11548.9 |  |
| 4-Hydroxybenzeneacetic acid | 152.0 | C8H8O3 | 559.3 |  |
| p-Hydroxybenzaldehyde | 122.0 | C7H6O2 | 2448.3 |  |
| p-Hydroxyacetophenone | 136.0 | C8H8O2 | 1127.8 |  |
| m-Hydroxybenzoic acid | 138.0 | C7H6O3 | 1974.0 |  |
| p-Hydroxybenzoic acid | 138.0 | C7H6O3 | 3034.3 |  |
| 3,5-Dihydroxybenzoic acid | 154.0 | C7H6O4 | 2345.3 |  |
| Vanillin | 152.0 | C8H8O3 | 5293.4 |  |
| Acetovanillone | 154.0 | C8H10O3 | 2430.0 |  |
| Vanillic acid | 168.0 | C8H8O4 | 4608.0 |  |
| Syringaldehyde | 182.0 | C9H10O4 | 4969.7 |  |
| Acetosyringone | 196.0 | C10H12O4 | 2763.1 |  |
| Syringic acid | 198.0 | C9H10O5 | 2678.2 |  |
| p-Coumaric acid | 164.0 | C9H8O3 | 1750.2 |  |
| Ferulic acid | 194.0 | C10H10O4 | 407.6 |  |
| 1,2-Benzenedicarboxylic acid | 166.0 | C8H6O4 | 1011.3 |  |
| Total |  |  | 50964.4 |  |
|  |  | V | 12331.4 |  |
|  |  | S | 10411.0 |  |
|  |  | C | 2157.8 |  |
| Diacids and hydroxy acids |  |  |  |  |
| 2-Hydroxypropanoic acid | 90.0 | C3H6O3 | 1085.3 |  |
| 2-Ketoisocaproic acid | 140.0 | C6H10O3 | 2851.5 |  |
| 2-Methyl-2-hydroxypropanoic acid | 104.0 | C4H8O3 | 2053.7 |  |
| 2-Methyl-2-hydroxybutanoic acid | 118.0 | C5H10O3 | 231.2 |  |
| 4-Methyl-2-hydroxypentanoic acid | 132.0 | C6H12O3 | 296.6 |  |
| 2-Hydroxy-2-pentenedioic acid | 146.0 | C5H6O5 | 192.0 |  |
| 3-Hydroxybutanoic acid | 104.0 | C4H8O3 | 380.0 |  |
| 2-Hydroxybutanedioic acid | 134.0 | C4H6O5 | 520.0 |  |
| 2-Hydroxypentanedioic acid | 148.0 | C5H8O5 | 476.0 |  |
| 2-Hydroxyhexanedioic acid | 162.0 | C6H10O5 | 729.2 |  |
| Ethanedioic acid | 90.0 | C2H2O4 | 6077.5 |  |
| Propanedioic acid | 104.0 | C3H4O4 | 170.6 |  |
| Succinic acid | 118.0 | C4H6O4 | 2897.1 |  |
| 2-Methylbutanedioic acid | 132.0 | C5H8O4 | 1639.7 |  |
| Fumaric acid | 116.0 | C4H4O4 | 22096.7 |  |
| Methylmaleic acid | 130.0 | C5H6O4 | 776.9 |  |
| Heptanedioic acid | 160.0 | C7H12O4 | 340.7 |  |
| Octanedioic acid | 174.0 | C8H14O4 | 814.9 |  |
| Azelaic acid | 188.0 | C9H16O4 | 1800.1 |  |
| Sebacic acid | 202.0 | C10H18O4 | 459.4 |  |
| Total |  |  | 45889.0 |  |

**Time (min)**

**#4**

**#2**

**# 1**

**MAG18**

**MAG16**

**st4**

**st3**

**st1**

*** 31**

*** 29**

*** 27**

*** 25**

*** 24**

*** 23**

*** 22**

*** 21**

*** 19**

*** 16**

**+ iso17**

**+ iso16**

**o 32**

**o 30**

**o 28**

**o 26**

**o 24**

**o 18**

**+ iso14**

**+ 30**

**+ 28**

**+ 26**

**+ 23**

**+ 22**

**+ 21**

**+ 20**

**+ 18**

**+18:1**

**+18:2**

**+ 17**

**+ 16**

**+16:1**

**+ 15**

**+ 14**

**+ 12**

**+ 9**

**+ 10**

**20**

**25**

**30**

**35**

**40**

**45**

**UK**

**#4**

**#3**

**#2**

**#1**

**MAG 18**

**MAG 16**

**st4**

**st3**

**st2**

**st1**

*** 31**

*** 29**

*** 27**

*** 25**

*** 24**

*** 23**

*** 22**

*** 21**

*** 18**

**+iso17**

**+iso16**

**+iso14**

**+ 30**

**+ 28**

**+ 26**

**+ 23**

**+ 22**

**+ 21**

**+ 20**

**+ 18**

**+18:1**

**+18:2**

**+ 17**

**+ 16**

**+16:1**

**+ 15**

**+ 14**

**+ 12**

**+ 10**

**+ 9**

**o 32**

**o 30**

**o 28**

**o 26**

**o 24**

**o 18**

**+ 24**

**+ 16**

**UK**

**O 32**

**MAG 18**

**MAG 16**

*** 27**

*** 25**

*** 24**

*** 22**

**+ 18**

**+ 14**

**O18**

**TIC of solvent extraction for NS**

**TIC of solvent extraction for TD**

**TIC of solvent extraction for TP**

Figure S1. The total ion chromatogram (TIC) of the solvent extraction of three soils.

+: n-Alkanoic acid; **O**: n-Alkanols; **＊**: n-Alkanes; **#**: carbohydrates; MAG: monoacylglycerides; st1–st4: steroids (see Table S1); UK: unknowns.

**Butylated hydroxytoluene**

**TIC for CuO oxidation of TD**

**Time (min)**

**TIC for CuO oxidation of TP**

**TIC for CuO oxidation of NS**

**Benzoic acid**

**Benzeneacetic acid**

**p-Hydroxybenzaldehyde**

**m-Hydroxybenzoic acid**

**p-Hydroxybenzoic acid**

**Vanillin**

**Vanillic acid**

**Syringaldehyde**

**Syringic acid**

**p-Coumaric acid**

**3,5-Dihydroxybenzoic acid**

**1,2-Benzenedicarboxylic acid**

**Succinic acid**

**Methylmaleic acid**

**UK6**

**p-Hydroxybenzaldehyde**

**Succinic acid**

**Azelaic acid**

**Fumaric acid**

**1,2-Benzenedicarboxylic acid**

**m-Hydroxybenzoic acid**

**Hydroxybenzeneacetic acid**

**1,3-benzenediol**

**Benzeneacetic acid**

**Benzoic acid**

**Butylated hydroxytoluene**

**p-Hydroxyacetophenone**

**p-Hydroxybenzoic acid**

**Vanillin**

**Acetovanillone**

**Vanillic acid**

**Syringaldehyde**

**Acetosyringone**

**Syringic acid**

**p-Coumaric acid**

**Ferulic acid**

**3,5-Dihydroxybenzoic acid**

**Methylmaleic acid**

**UK8**

**UK4**

**Butylated hydroxytoluene**

**UK6**

**10**

**15**

**20**

**25**

**30**

**35**

**Acetosyringone**

**Hydroxybenzeneacetic acid**

**Benzoic acid**

**Benzeneacetic acid**

**Succinic acid**

**p-Hydroxybenzaldehyde**

**Methylmaleic acid**

**p-Hydroxyacetophenone**

**Butylated hydroxytoluene**

**Vanillin**

**m-Hydroxybenzoic acid**

**Acetovanillone**

**1,2-Benzenedicarboxylic acid**

**Syringaldehyde**

**Vanillic acid**

**Azelaic acid**

**p-Coumaric acid**

**Syringic acid**

**Ferulic acid**

**UK8**

**UK6**

**UK4**

**Fumaric acid**

Figure S2. The total ion chromatogram (TIC) of CuO oxidation products of the three soils.

**TIC for TD**

**6**

**8**

**10**

**12**

**14**

**16**

**18**

**20**

**22**

**24**

**26**

**8**

**7**

**6**

**5**

**4**

**3**

**2**

**1**

**6**

**8**

**10**

**12**

**14**

**16**

**18**

**20**

**22**

**24**

**26**

**TIC for TP**

**8**

**7**

**6**

**5**

**4**

**3**

**2**

**1**

**6**

**8**

**10**

**12**

**14**

**16**

**18**

**20**

**22**

**24**

**26**

**TIC for NS**

**8**

**7**

**6**

**5**

**4**

**3**

**2**

**1**

**15**

**16**

**17**

**18**

**19**

**8**

**7**

**6**

**5**

**4**

**Time**

Figure S3. The total ion chromatogram (TIC) of BPCAs in three soils.
